# Expert opinions and clinical experiences with chlormethine gel as maintenance treatment for patients with mycosis fungoides

**DOI:** 10.3389/fmed.2023.1298988

**Published:** 2024-01-18

**Authors:** Larisa Geskin, Christiane Querfeld, Emmilia Hodak, Neda Nikbakht, Evangelia Papadavid, Marco Ardigò, Ulrike Wehkamp, Martine Bagot

**Affiliations:** ^1^Columbia University Medical Center, Columbia University, New York, NY, United States; ^2^Division of Dermatology and Department of Pathology, City of Hope National Medical Center and Beckman Research Institute, Duarte, CA, United States; ^3^Davidoff Medical Center, Rabin Medical Center, Tel Aviv University, Tel Aviv, Israel; ^4^Department of Dermatology and Cutaneous Biology, Thomas Jefferson University, Philadelphia, PA, United States; ^5^National Center of Excellence for Rare Disease, Second Department of Dermatology and Venereology, Attikon University General Hospital, Athens, Greece; ^6^San Gallicano Dermatological Institute, IRCCS, Rome, Italy; ^7^Dermatology Unit, IRCCS Humanitas Research Hospital, Rozzano, Italy; ^8^Department of Biomedical Sciences, Humanitas University, Pieve Emanuele, Italy; ^9^Department of Dermatology, University Hospital Schleswig-Holstein, Kiel, Germany; ^10^MSH Medical School Hamburg, Hamburg, Germany; ^11^Department of Dermatology, AP-HP, Université de Paris, Hôpital Saint-Louis, Paris, France

**Keywords:** mycosis fungoides, chlormethine gel, maintenance therapy, expert opinions, clinical experiences

## Abstract

Maintenance treatment can be recommended for patients with mycosis fungoides (MF) whose disease responds to primary treatment. While positive outcomes have been observed in small studies with maintenance therapy, there is a lack of practical guidelines and agreement on when and how maintenance therapy for MF should be approached. In this article, we discuss expert opinions and clinical experiences on the topic of maintenance therapy for patients with MF, with a focus on chlormethine gel. Ideally, patients should have a durable response before initiating maintenance therapy. The definition of and required duration of durable response are topics that are open to debate and currently have no consensus. Chlormethine gel has several attributes that make it suitable for maintenance therapy; it can be easily applied at home, can be combined with other treatment options for maintenance, and has a manageable safety profile. Chlormethine gel as maintenance therapy can be applied at decreasing frequencies after active treatment with chlormethine gel or other therapies until the minimally effective dose is reached. Patients generally tend to adhere well to chlormethine gel maintenance regimens and may remain on treatment for several years. The experiences described here may be useful for clinicians when deciding on maintenance treatment regimens for their patients. Development of guidelines based on clinical trial outcomes will be important to ensure the most effective maintenance treatment strategies are used for patients with MF.

## Introduction

Mycosis fungoides (MF), the most common form of cutaneous T-cell lymphoma, is a rare disease that generally presents with patches or plaques on the skin (1, 2). MF has a slowly progressive, chronic clinical course (3). While patients may achieve complete or partial remission after initial treatment, most will experience disease recurrence and a few will eventually have disease progression (4). The use of maintenance treatment could potentially prevent or delay recurrence or even progression. The National Comprehensive Cancer Network guidelines for primary cutaneous lymphoma indicate that therapies with lower side-effect profiles and no cumulative toxicity can be given in an ongoing or maintenance fashion to maintain disease control and quality of life (QOL) (5).

Several small studies have reported positive outcomes with maintenance therapy for patients with early/late-stage MF. In a retrospective evaluation of 14 patients with early-stage MF (*n* = 10 stage IA–IB; *n* = 4 stage IIA) treated with narrowband UV B (nbUVB), no relapses were seen for 18 months among eight patients with a complete response (CR) who received the recommended nbUVB maintenance protocol (6). A prospective trial randomized 19 patients with early-stage (IA–IIA) MF who had a CR after psoralen and UV A (PUVA) induction to PUVA maintenance for 9 months or no maintenance. Use of maintenance therapy extended median disease-free remission from 4 (1–20) to 15 (1–54) months (7). Another prospective study comparing total skin electron beam therapy (TSEBT) treatment with/without subsequent bexarotene maintenance, included patients with early- (*n* = 15) and late-stage MF (*n* = 31) as well as Sézary syndrome (*n* = 7). Progression-free survival was 17 months with bexarotene maintenance vs. 5 months without maintenance (8). Finally, a single-center experience reported long-term (>1 year) disease control in three patients with late-stage MF who continued to receive doxorubicin following an initial response (9).

While maintenance therapy is usually recommended, the lack of practical guidelines and agreement on when and how maintenance therapy for MF should be approached can pose challenges for clinicians in determining the best course of action. In addition, comparison between studies on maintenance regimens is difficult due to variation in study populations and treatments. Even studies on the same treatment can be hard to compare as differences in parameters, such as follow-up duration, definition of relapse-free interval, and definition of relapse, are not always consistent across studies (10). Herein, we discuss expert opinions and clinical experiences on the topic of maintenance therapy for patients with MF, with a focus on chlormethine gel.

## Maintenance treatment for patients with MF

The main goal for patients with MF undergoing maintenance therapy is to sustain their current response with an easily implemented regimen once the response is considered acceptable, whether this is CR, very good partial response (VGPR), or partial response (PR). VGPR has been defined as 75–<100% reduction from baseline in Composite Assessment of Index Lesion Severity, in Modified Severity-Weighted Assessment Tool, or of body surface area (11).

One important unanswered question regarding maintenance therapy for MF involves the most effective time to initiate the regimen. If maintenance therapy is begun before the optimal response is reached, there is a risk for relapse. Ideally, patients should have a durable response before starting maintenance. However, the definition of a durable response is a contentious issue for which there is currently no consensus. Here, we present a several suggestions and considerations on how durable response may be defined; however, these suggestions must be verified and confirmed by data. Achieving CR can be more difficult than achieving (VG)PR in patients with MF; therefore, the time needed for a CR to be considered durable may be shorter than that required for a (VG)PR to be so considered. Due to differences in disease severity, durable response may need to be defined differently for patients with early- and late-stage disease, with a decreasing time interval with increasing disease severity; in general, during late-stage disease, a response could be considered durable earlier than for patients with early-stage disease. The overall survival of patients with early-stage disease is not impacted as greatly as during late-stage disease, and longer treatment times may be tolerated while awaiting response. Currently, there is no real consensus on when a response should be considered durable during early-stage disease; especially for CR. United States Cutaneous Lymphoma Consortium guidelines for phototherapy indicate a 1-month period of continued clearance is needed to document a CR before considering maintenance therapy (12). Our overall suggestion is to consider a VGPR durable after 4–6 months and a PR durable after 6–12 months.

While it is clear that patients need to have a durable response before considering maintenance therapy, another open question is how long they should be in durable remission before moving to maintenance therapy. Achieving a durable response does not mean patients should start maintenance therapy straightaway. During early-stage MF, maintenance therapy is not a rule, and many patient- and disease-related factors influence whether a maintenance phase is initiated. In contrast, in late-stage disease, maintenance therapy is required, given the high likelihood for relapse, which can greatly impact prognosis and QOL. Therefore, there is a higher need for certainty about the response in these patients and a longer duration of durable response may be required for patients with late-stage disease before starting maintenance therapy.

Once initiated, there are no clear directions on how long maintenance therapy should continue. While patients with late-stage disease require more-frequent follow-up to maintain stable response, the duration of maintenance therapy in MF irrespective of stage may depend on age of the patient, disease stage, type of lesions, type of therapy, and treatment history. Ideally, maintenance therapy should last indefinitely, but often this is not possible due to treatment fatigue, adverse events (AEs), or insurance problems.

## Chlormethine gel as maintenance treatment for MF

The ideal maintenance treatment should be easy to administer, safe to use long-term without significant AEs, and cost-effective.

Chlormethine (also known as mechlorethamine) has been used as topical treatment for MF for many years. It was initially available in aqueous-based and compounded ointment-based formulations (13, 14) and a limited number of studies have investigated maintenance treatment with these chlormethine formulations (15). In a retrospective analysis of 148 patients with MF, patients who received aqueous- or ointment-based chlormethine following TSEBT maintained a higher rate of relapse-free survival than patients who received no maintenance (16). A combined modality study investigated a treatment regimen of interferon-α and isotretinoin followed by TSEBT and long-term maintenance with topical chlormethine between 1987 and 2001. Among 38 patients with early-stage MF, a median disease-free survival of 62 months was seen with this regimen (17). A single-center, retrospective cohort study investigated 203 patients with MF who received aqueous or ointment-based chlormethine as initial therapy. Among 81 patients who had various durations of maintenance therapy with chlormethine, results showed that CR was better maintained during the maintenance phase, although patients relapsed at a similar rate after therapy ended (18). Neither the aqueous- or ointment-based formulations of chlormethine were approved by the US Food and Drug Administration for treatment of MF, despite positive clinical outcomes (18). Moreover, patients were reported to experience difficulties with preparation and application of these formulations. Therefore, a novel CL gel formulation was developed.

Chlormethine 0.016% w/w topical gel (equivalent to 0.02% chlormethine hydrochloride) is approved as monotherapy in the United States (19) and Israel (20) for treatment of patients with early-stage (IA–IB) MF who received prior skin-directed therapy, and in the European Union (21) for treatment of adult patients with early-stage MF. The gel formulation is nongreasy and quick drying, making it easy to apply at home by patients or caregivers. AEs seen with chlormethine gel treatment are generally skin related and often mild (22), and systemic AEs are unlikely to occur as there is no evidence of absorption of chlormethine after topical administration (23). High response rates were seen with active chlormethine gel treatment in the pivotal trial and various real-world studies (24–28).

While chlormethine gel is currently used in clinical practice as maintenance therapy, clear guidelines for this treatment in a maintenance setting are lacking. Data from the United States-based prospective, observational PROVe study, which assessed chlormethine gel in real-world clinical practice, showed that the majority of patients (91%) continued using chlormethine gel in a maintenance setting (3). Maintenance therapy was defined as continued chlormethine gel use after achieving at least PR, and most patients maintained their once-daily treatment schedule. Chlormethine gel treatment continued for ≥6 months in 40% of patients and ≥ 12 months in 22% of patients. A retrospective study of patients with MF treated at Thomas Jefferson University between 2012 and 2020 investigated progression-free survival and efficacy of chlormethine gel in patients using it as active treatment or for maintenance after TSEBT (29). Twenty-three patients received maintenance therapy and had a progression-free survival rate of 65% with median time to progression of 29 months. In addition, patients reported improved QOL while receiving maintenance therapy.

## Expert opinions and clinical experiences with chlormethine gel maintenance therapy

Initiating maintenance therapy is an individual decision that treating clinicians should make in agreement with each patient. On the basis of our clinical expertise and experience, we believe chlormethine gel should be considered as a maintenance therapy option after patients achieve a durable CR, VGPR, or PR. In most patients, it will likely be appropriate to reduce the application frequency of chlormethine gel when the patients move from active to maintenance treatment. The dosing schedule may depend on the original treatment regimen used before achieving response. When patients had used chlormethine gel daily as active treatment, keeping the same frequency for maintenance therapy is generally not recommended, although it may be appropriate in individual cases with a (VG)PR. The application frequency may be reduced slowly, changing every ~1–2 months from every other day, to three times per week, two times per week, once weekly, and then with further decreases in frequency. If a patient has disease progression at any time during dose reduction, further treatment will be needed.

Chlormethine gel is also a feasible maintenance treatment for patients who used other therapies to achieve a durable response. A *post hoc* analysis of the pivotal trial data showed that the response to chlormethine gel was similar in patients with MF who received prior treatment with bexarotene/phototherapy vs. other therapies, indicating that chlormethine gel is a valid treatment option for patients who received prior therapies (30). Chlormethine gel may also be considered after skin-directed therapies other than phototherapy, such as TSEBT or localized radiation, and after various systemic therapies other than bexarotene, such as interferon, methotrexate, histone deacetylase inhibitors, brentuximab vedotin, or immunotherapy.

Considerations for use of chlormethine gel as maintenance therapy, based on clinical experiences, are presented in Table 1. Certain clinical and personal circumstances may result in a preference for chlormethine gel over other options. For example, patients may have experienced AEs with other maintenance options or have contraindications to treatment. In addition, lesions may be present in locations difficult or impossible to reach with phototherapy. Patients can also have personal schedules that do not allow for travel to receive phototherapy, or may have a preference for a particular treatment. Chlormethine gel dosing during maintenance therapy can be influenced by the extent and location of disease, lesion type, response (CR, VGPR, or PR), and chlormethine gel treatment frequency patients were using when they achieved the best response. Ideally, patients should be treated with the minimally effective dose to balance QOL and disease stability or remission.

**Table 1:** Considerations for chlormethine gel therapy focused on the maintenance phase, based on authors’ clinical experiences.

Circumstances or clinical presentations for which chlormethine gel may be preferred as a skin-directed therapy over other treatments	Patients with a contraindication to or side effects with other therapies Lesions in locations that cannot be reached by ultraviolet light, the most frequently used skin-directed therapy for MF Patients who cannot travel to receive phototherapy (due to work or other personal circumstances) Patients with a preference for certain treatment type
Factors influencing chlormethine gel dosing or decreases in dosing during maintenance treatment	Extent of disease Location of lesions Lesion type Residual patches Plaques – often more resistant to treatment and can recur Response type (CR or PR) Treatment frequency when best response was achieved
Adherence to chlormethine gel maintenance therapy	Generally, patients adhere well to chlormethine gel maintenance Consider the patient’s opinion when deciding if maintenance treatment should continue or end Educate patient on expectations of disease (chronic and recurrent) and treatment (skin toxicities) Some patients may not always adhere to the maintenance regimen, even when dosing is reduced
Duration of chlormethine gel maintenance therapy	Approximately 1–2 years; however, this can range from months to years depending on disease characteristics If CR is maintained on lowest-frequency treatment, it may be possible to stop maintenance, again depending on patient characteristics In case of PR or frequent relapses, long-term maintenance can be necessary
Chlormethine gel combination therapy for maintenance	Chlormethine gel maintenance can be combined with topical steroids Systemic retinoids can be added to reduce the frequency of chlormethine gel treatment Combinations used in clinical practice for patients with more-advanced or resistant MF are chlormethine gel with methotrexate, oral bexarotene, romidepsin, or mogamulizumab^a^
Reasons for discontinuation of chlormethine gel as maintenance treatment	Sustained CR Skin toxicity Treatment fatigue Costs associated with chlormethine gel Insurance coverage changes Patient wants to pause or stop treatment Disease progression Other, including plans for pregnancy

^a^No combinations have been approved and these must be carefully considered for individual cases. CR, Complete response; MF, Mycosis fungoides; and PR, Partial response.

In our experience, patients generally adhere very well to chlormethine gel maintenance regimens. To further improve adherence, it is important to consider the patient’s opinion when deciding if maintenance treatment should continue or end, and to educate patients on expectations of disease (chronic and recurrent) and treatment (skin toxicities). An aggressive moisturization and proper skin care routine, such as the Geskin regimen (31), can also help patients to continue therapy.

In certain cases, it may be effective to combine chlormethine gel with other maintenance treatments, for example topical corticosteroids. Systemic retinoids can be added to the maintenance regimen, which sometimes allows for reduced frequency of chlormethine gel application. For patients with refractory early-stage or more advanced MF, maintenance treatment with chlormethine gel may be combined with mogamulizumab, methotrexate, oral bexarotene, or romidepsin. However, these combinations are not approved and must be carefully considered for individual cases.

On average, patients remain on chlormethine gel maintenance therapy for ~1–2 years; however, this period can range from months to multiple years depending on disease characteristics. It may be possible to stop maintenance therapy if a patient has a maintained CR on low treatment frequency, but this is also dependent on patient characteristics. In cases of PR or frequent relapses, long-term maintenance may be required. Patients who discontinue chlormethine gel maintenance for reasons other than CR tend to do so because of AEs, treatment fatigue, costs or changes in insurance coverage, a desire to pause or stop treatment, or disease progression.

## Patients receiving chlormethine gel as maintenance in clinical practice

To provide real-world examples of patients receiving chlormethine gel maintenance therapy, we briefly discuss two cases.

**Figure 1:**
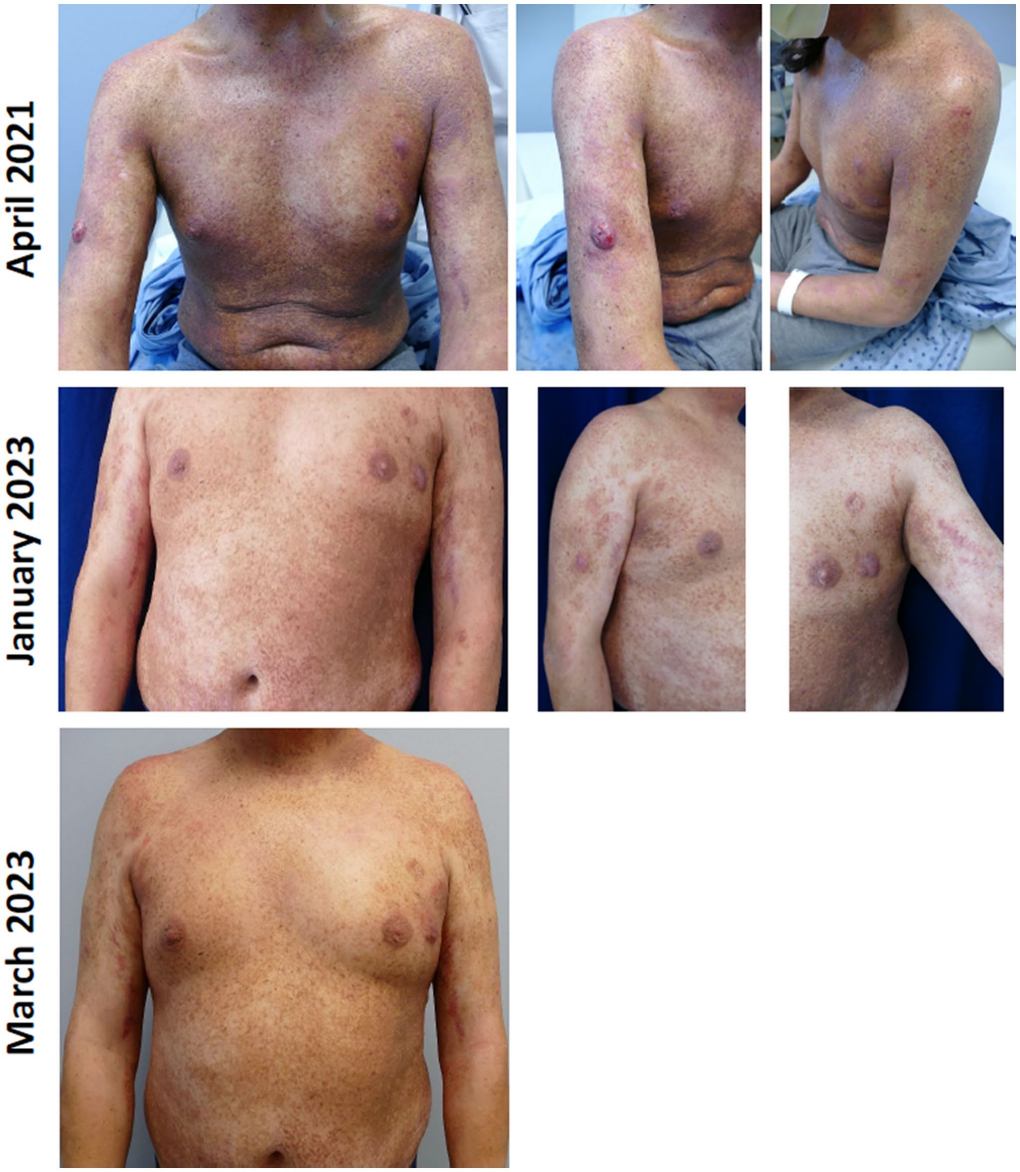
Patient with stage IIB mycosis fungoides treated with chlormethine gel as maintenance therapy.

The first patient was a 37-year-old man with stage IIB MF (T3N0M0B0a) receiving systemic oral bexarotene combined with nbUVB thrice weekly for poikilodermatous patches, erythematous plaques, and scattered tumors without histopathologic evidence for large cell transformation. The patient had received intermittent palliative radiation treatment to scattered tumor nodules. His response to phototherapy had plateaued and he developed numerous new plaques and a few tumor nodules. Due to work and personal preference, the patient did not desire other infusion therapies or to switch treatments, and chlormethine gel maintenance therapy three times per week was initiated. All plaque and tumor lesions were treated with chlormethine gel and improved during maintenance therapy, including the tumor lesions (Figure 1). Chlormethine gel was increased to five times per week. The patient has continued chlormethine gel treatment for the last 3 years without significant AEs. He experienced only mild to moderate erythema and pruritus overlying treated lesions.

The second patient was a 74-year-old man with stage IA MF who had a near-CR with chlormethine gel treatment while using it daily in conjunction with topical corticosteroids three times per week. Considering his excellent response, therapy was tapered to three times per week and then to once weekly as he maintained his near-CR. At this point, maintenance therapy with biweekly application of chlormethine gel without topical corticosteroids was initiated. During maintenance treatment with chlormethine gel, the patient developed dermatitis in the gel application areas, mostly the skin folds. The patient believed this to be disease progression. However, on physical examination, the patient’s skin revealed well-demarcated erythematous patches, which clearly followed his pattern of gel application. Consequently, he was diagnosed with mild contact dermatitis and chlormethine gel was paused for 2 weeks, while the patient used mid-potency topical corticosteroids twice daily. The dermatitis responded well to treatment with only mild post-inflammatory hyperpigmentation in affected areas. A discussion was held with the patient regarding maintenance therapy in light of the mild dermatitis, and risks and benefits of observation only (without maintenance therapy) were explained. The patient elected to continue maintenance therapy to the affected areas biweekly followed by topical corticosteroid application on the following day. The dermatitis did not recur; the patient remains in near-CR for over 12 months and continues on maintenance therapy.

## Discussion

Most patients with early-stage MF and almost all patients with advanced-stage MF will have recurrence/disease progression after achieving remission (4). While some guidelines recommend patients with MF, who respond to primary treatment should be considered for maintenance treatment, there is no clear direction on how and when to start patients on a maintenance regimen (5, 32). As a result, clinicians may be unsure how to best treat patients in this setting. No studies have investigated the best timing, best treatment type, and needed duration of maintenance therapy for patients with MF to date. A number of small studies have described positive effects with maintenance regimens using phototherapy in patients with early-stage MF (6, 7). However, maintenance treatment with phototherapy is still contested, since positive results on disease-free remission intervals have only been described in small studies without statistical significance (10). In addition, a study with 30 patients with early-stage MF saw no significant association between recurrence-free survival in patients who received maintenance phototherapy and patients who did not for both PUVA (*p* = 0.63) and nbUVB (*p* = 0.3) (33). Other small studies demonstrated positive outcomes with maintenance therapy using bexarotene or doxorubicin (8, 9); however, these results were limited and must be confirmed in larger studies. All treatments can also potentially be harmful and result in AEs, making it vital to carefully weigh risks and benefits of any treatment regimen, in particular for systemic maintenance options.

In this article, we describe several considerations for use of chlormethine gel as maintenance therapy for patients with MF on the basis of expert opinions and clinical experiences. These experiences may be helpful for clinicians when deciding on maintenance treatment regimens for their patients.

The previously used aqueous-based and compounded ointment-based formulations of chlormethine were already used as maintenance treatment for patients with MF (15), resulting in improved relapse-free or disease free-survival (16–18). The novel gel formulation has also been used in clinical practice as a maintenance therapy, with positive effects on progression-free survival and QOL (29). Real-world data showed that most patients continued using chlormethine gel after a PR (3). While no guidelines are available for maintenance treatment with chlormethine gel, this treatment option does have several attributes that may make it particularly suitable for maintenance therapy for patients with MF. The topical treatment can easily be applied by patients at home, thereby avoiding additional clinic or hospital visits for which they may not have time or are unwilling to attend, especially after achieving remission. For patients with more-advanced or resistant disease, chlormethine gel maintenance can be combined with other treatment options without concerns about drug–drug interactions (23). Chlormethine gel has a manageable safety profile (22), which is important for maintenance treatments. AEs that have been observed with chlormethine gel were all skin related (24, 25). Contact dermatitis is the AE most likely to lead to treatment discontinuation; however, many patients can continue or reinitiate chlormethine gel treatment with proper management (26, 34).

While maintenance treatment decisions depend on a given patient’s disease status, the development of general guidelines for clinicians would be very useful. These guidelines, outlining when maintenance regimens should be initiated, would benefit greatly from a consensus on the classification/definition of a durable response. Definitions currently tend to vary by institution and depend on clinical scenarios. Durable response may be different for patients with early- and late-stage disease and a CR may be considered durable earlier than (VG)PR. In addition, clearer definitions of maintenance treatment in general and time to next treatment, as well as criteria to enable comparison of disease management and potential benefits, are urgently needed. Ideally, a prospective study should be undertaken to investigate the efficacy of maintenance therapies for patients with MF. Such a trial must be carefully designed to overcome the difficulties presented by lack of clear definitions and would require complex inclusion criteria.

In conclusion, maintenance therapy is vital for many patients with MF, but lack of consensus on definitions and scarcity of data on the topic can complicate clinicians’ determination of when and how patients should be treated. The development of more-detailed guidelines will be important to ensure the most effective maintenance treatment strategies are used for patients with MF.

## Data availability statement

The original contributions presented in the study are included in the article/supplementary material, further inquiries can be directed to the corresponding author.

## Ethics statement

Written informed consent was obtained from the individual(s) for the publication of any potentially identifiable images or data included in this article.

## Author contributions

LG: Conceptualization, Writing – original draft, Writing – review & editing. CQ: Conceptualization, Writing – original draft, Writing – review & editing. EH: Conceptualization, Writing – original draft, Writing – review & editing. NN: Conceptualization, Writing – original draft, Writing – review & editing. EP: Conceptualization, Writing – original draft, Writing – review & editing. MA: Conceptualization, Writing – original draft, Writing – review & editing. UW: Conceptualization, Writing – original draft, Writing – review & editing. MB: Conceptualization, Writing – original draft, Writing – review & editing.
